# Development of a novel dynamic nosocomial infection risk management method for COVID-19 in outpatient settings

**DOI:** 10.1186/s12879-024-09058-w

**Published:** 2024-02-19

**Authors:** Yuncong Wang, Lihong Wang, Wenhui Ma, Huijie Zhao, Xu Han, Xia Zhao

**Affiliations:** Hospital Infection Management Division, Xuanwu Hospital Capital Medical University, No. 45 ChangChun Street, Xicheng District, Beijing, 100053 People’s Republic of China

**Keywords:** COVID-19, ARIMA model, Infection control, Outpatient Clinics

## Abstract

**Background:**

Application of accumulated experience and management measures in the prevention and control of coronavirus disease 2019 (COVID-19) has generally depended on the subjective judgment of epidemic intensity, with the quality of prevention and control management being uneven. The present study was designed to develop a novel risk management system for COVID-19 infection in outpatients, with the ability to provide accurate and hierarchical control based on estimated risk of infection.

**Methods:**

Infection risk was estimated using an auto regressive integrated moving average model (ARIMA). Weekly surveillance data on influenza-like-illness (ILI) among outpatients at Xuanwu Hospital Capital Medical University and Baidu search data downloaded from the Baidu Index in 2021 and 22 were used to fit the ARIMA model. The ability of this model to estimate infection risk was evaluated by determining the mean absolute percentage error (MAPE), with a Delphi process used to build consensus on hierarchical infection control measures. COVID-19 control measures were selected by reviewing published regulations, papers and guidelines. Recommendations for surface sterilization and personal protection were determined for low and high risk periods, with these recommendations implemented based on predicted results.

**Results:**

The ARIMA model produced exact estimates for both the ILI and search engine data. The MAPEs of 20-week rolling forecasts for these datasets were 13.65% and 8.04%, respectively. Based on these two risk levels, the hierarchical infection prevention methods provided guidelines for personal protection and disinfection. Criteria were also established for upgrading or downgrading infection prevention strategies based on ARIMA results.

**Conclusion:**

These innovative methods, along with the ARIMA model, showed efficient infection protection for healthcare workers in close contact with COVID-19 infected patients, saving nearly 41% of the cost of maintaining high-level infection prevention measures and enhancing control of respiratory infections.

## Background

During the coronavirus disease 2019 (COVID-19) pandemic, many countries experienced peaks of infection, which were associated with serious social and economic consequences [1]. The world health organization (WHO), which declared the COVID-19 pandemic in March 2020, has estimated that, up to 12 October 2023, there had been 771,191,203 confirmed cases of COVID-19 worldwide, including 6,961,014 deaths, with the highest daily number of newly confirmed cases being 44,236,225 on 19 December 2022 [2].

Unlike previously identified viruses such as severe acute respiratory syndrome (SARS) and middle east respiratory syndrome (MERS) viruses, the virus responsible for COVID-19 has a high mutation frequency and a high transmission capacity (R_0_). The R_0_ of COVID-19 has been estimated to average 3.3 (range, 1.4–6.5) [3, 4], and its mutation capacity was estimated to be approximately 1.1*10^3^ substitutions per site per year, or nearly one substitution every 11 days [5]. These characteristics have increased the difficulty of recognizing and diagnosing COVID-19, and reduced the immunoprotection provided by vaccines, thus limiting infection control in hospitals. Epidemiological concerns, the capacity of the health system, new infection control methods and economic feasibility should all be considered in developing policies and management plans to control COVID-19 [6].

Airborne infection, which contributes to 10% to 20% of endemic nosocomial infections in hospitals, is a core problem in infection control in outpatients [7]. Increases in the risk of transmission of respiratory infectious diseases in outpatients have been associated with higher demands for medical care, greater severity of the treated conditions, and increases in the complexity of the procedures performed in outpatient settings [8, 9]. Moreover, limitations in infection prevention infrastructure and resources [8], as well as inadequate compliance with infection prevention and control measures, can further increase risks of infection among patients and healthcare workers (HCWs) [10]. Most COVID-19 outpatients have displayed symptoms that were of similar or less severe intensity than those of influenza [11], thus complicating the recognition of COVID-19 infection in outpatients.

It may be more challenging to identify outpatients with COVID-19 in China than in other countries because of the lack of surveillance capacity and the insufficiency of resources for preventing infection in outpatients [12]. Identifying infected outpatients may also be complicated by the presence in populations of multiple genetic variants, high viral transmission, and the seasonal prevalence of COVID-19 [13, 14]. A management method that can predict the risk of infection is essential to improve the quality of COVID-19 infection control in outpatients [15].

Advances in prediction models, artificial intelligence and other computer technologies have enhanced clinical diagnoses, pathogen classification and estimates of epidemic trends of infectious diseases [16–18]. The accuracy and efficiency of these methods have provided important technical support for the prevention and control of respiratory infectious diseases, such as COVID-19 [19]. The auto regressive integrated moving average (ARIMA) model is currently the most widely used model for predicting outcomes [20], with its use of time-series analysis having better ability to predict periodic changes and other related random variables. This model has been widely used in various fields, including economics [21] and demography [22]. This model has also shown the ability to accurately predict seasonal outbreaks of infectious diseases, including COVID-19 [23, 24], and to estimate the number of infected patients and the severity of the COVID-19 epidemic [25]. Epidemiological data from the Johns Hopkins Medical Center have been used to predict the prevalence and incidence of COVID-19 [26]. Moreover, the ARIMA model has been used to generate short-term (10-day) forecasts of the number of daily confirmed cases of COVID-19 in Canada, France, India, South Korea, and the UK [27] and to predict COVID-19 trends in Italy [28] and Canada [29]. Implementation of deep learning techniques and artificial Intelligence in the ARIMA model has yielded accurate forecasts of COVID-19 infection and severity [30, 31].

The present study describes the development of a method of infection prevention management for outpatients by combining hierarchical respiratory control measures with ARIMA-based early warning. This made the risk assessment of COVID-19 in outpatients more dynamic.

## Methods

### Study design and setting

This study included outpatients diagnosed with or treated for COVID-19 from 15 November 2022 to 23 May 2023 at Xuanwu Hospital Capital Medical University in China, a Grade three Class A comprehensive hospital with 1643 inpatient beds and over 8000 outpatient visits per day.

This retrospective study was performed to develop a new respiratory infection control method for COVID-19 in outpatients. To achieve this goal, the research was conducted by four steps. First, previously obtained surveillance data were fit to an ARIMA model to predict the possible values during the following week (the section of ARIMA model and the prediction process). Second, the risk threshold calculation method published by WHO was used as the standard to delimit the value range of low and high risk level for the predicted value (the section of The threshold of risk level). Third, relevant literatures, guidelines and national standards were searched systematically to summarize the measures for respiratory tract nosocomial infections prevent and control (the section of Literature selection and infection control measures extraction). Fourth, the appropriate measures were selected to reach an expert consensus using the Delphi method, and these measures were divided into two levels, corresponding to low risk level and the high risk level, to construct a hierarchical measures database. The expert panel also established the risk assessment criteria to determine which level of the measures should be adopted in the outpatient setting, according to the predicted value of ILI and BI data and the value range delimit by the second step (the section of The Delphi process). By these four steps, outpatient settings can achieve more dynamic and accurate COVID-19 infection control outcomes.

### Data source

This study utilized two data sources: weekly surveillance data on influenza-like illnesses (ILI) and COVID-19-associated search engine data. ILI was defined as an acute respiratory infection with a temperature higher than 38 °C and coughing that began within the previous ten days [32]. Search engine data, such as Google Trends data, have previously been used to investigate and assess the use of non-hospital data sources in forecasting infectious epidemics [33, 34]. Because the use of Google Trends data is restricted in mainland China, data were obtained from the Baidu Index (BI), downloaded from https://index.baidu.com/v2/index.html#/. The ILI and BI datasets reviewed in this study were collected from 1st January 2021 to 31st December 2022.

### ARIMA model and the prediction process

The ARIMA model developed by Box and Jenkins was utilized to predict future behavior. If numbers show some degree of correlation, a time series can forecast and estimate more accurately. Auto regression (AR), or the regression of previous values from time series, was one of the three parts of the model. A lagged observation-based moving average model is referred to as a moving average (MA). The function for enhancing the stability of time series is referred to as integrated (I). The ARIMA model fits data according to three parameters: p, d, and q; with p being the lag observations of the model, or lag order; d the number indicating how many times the raw observations were differentiated; and q the size of the window for the moving average. The ARIMA model can be summed up in the standard notation as ARIMA (p,d,q) [35].

In most studies, models are selected using the Akaike Information Criterion (AIC). The AIC should be as low as practical to keep the model as simple as possible while measuring the predictive accuracy of model fitting. The model with the lowest AIC was considered optimal based on the standard model selection method of the present study, which used AIC [36].

The R “forecast” package used maximum likelihood estimation (MLE) for model fitting. Graphs of the partial auto correlation function (pacf) and the auto correlation function (acf) enabled identification of the parameters of p and q. The model with the lowest AIC score was subsequently used for value forecasting. The complete model fitting calculation process was performed by R 4.0.3 with the help of the “ggplot” and “forecast” packages.

Model fitting was evaluated using the mean absolute percentage error (MAPE) criterion by comparing the predicted results with actual values. MAPE is a prediction-making technique that is regarded as more accurate than other methods, as it transforms absolute errors to percentages of actual numbers [36].

In this study, the data of ILI and BI was used to fit ARIMA model for prediction. Consecutive data of first 30 weeks (1st January 2021 to 31st July) was used as training sample to find the best model fitting parameters and perform prediction for the 31th week.

The rolling prediction was performed consecutively week by week in two time period, 8th September 2019 to 1st May 2020, and 1st August 2021 to 31st December 2022. The former one was for model fitting verification and the latter one was for the management practice. The consecutive prediction results and the actual data were collected during 1st August 2021 to 31st December 2022 to evaluate the accuracy of prediction by the MAPE.

### The threshold of risk levels

The infection warning threshold was established using the WHO method [32] as a criterion to differentiate between high and low risk periods for COVID-19 nosocomial infections in outpatient clinics. Because of its ease of use and the absence of seasonal dependency, the WHO approach was selected above other pandemic threshold techniques, including the moving epidemic method (MEM) and cumulative sum (CUSUM) techniques. The sample size for this study was 40 recent weekly data points, and the risk warning threshold was set as the mean ± the standard deviation (SD). The thresholds can be used to determine whether the predicted risk in the coming week exceeds the alert level.

### Literature review and infection control measures extraction

To extract sufficient infection control measures for COVID-19. experts on nosocomial infection control systematically searched the PubMed, MEDLINE, and WANFANG med online databases prior to the Delphi process, using the terms “COVID-19”, “infection control”, “outpatient”, “disinfection” and “personal protective equipment”. Guidelines and standards from the National Health Commission of the People’s Republic of China, the Standardization administration of China and the Centers for Disease Control and Prevention of the USA were also searched for suggestions on infection control measures and interventions.

Infection control measures were screened and extracted from 25th July to 30th November 2022 by two researchers, acting independently. Data extracted included standard/guideline/research name, date, location, organization, infection control measures, and infection control outcomes. The inclusion criteria for articles searching were: 1) published between 1st January 2010 to 31st December 2022; 2) reported on infection control measures against hospital-acquired respiratory infection; 3) written in English; and 4) full-text availability. The exclusion criteria for articles searching were: 1) unavailable full English texts; 2) comments, case reports, editorials, letters or conference summaries.

### The Delphi method and the construction of the risk assessment criterion

The Delphi method was used to select infection control measures from the literature review and divide into low and high risk levels to construct a hierarchical measures database. The risk assessment criterion, also constructed by the expert panel of Delphi method, made it possible to select appropriate level of the infection control measures to be adopted in the outpatient setting, according to the prediction results and the threshold of risk levels.

Using data searching and extraction, a panel of experts selected the infection control measures from the measures extracted of the literature review. The panel, which consisted of about 29 experts, included clinicians with expertise in clinical medicine, nursing, and nosocomial infection control. Experts were selected if they had at least an associate senior title, at least five years of clinical work experience, and high enthusiasm for participation. Finally, the research panel consisted of 13 clinical physicians, five nurses, seven experts on nosocomial infection control, two experts on outpatient management, and two experts on nursing management, including the chief of the Beijing Association of Preventive Medicine’s Hospital Infection Control Committee and the deputy chief of the Hospital Infection Management Committee of the Chinese Hospital Association.

Measures to attain consensus were selected using a two-round Delphi process. During the first round, the experts on the panel were asked to rate these measures by degree of importance on a five-point Likert scale using a questionnaire. Measures with scores over 3.9 were selected for the second round [37]. During the second round, the panelists were asked to “agree” or “disagree” with each measure, with those measures agreed to by at least two-thirds of these experts included [38].

In addition to screening prevention and control measures, the experts also constructed the risk assessment criterion. When the prediction results of ILI and BI data met the enabling conditions of the risk assessment criterion, the corresponding level of infection control measures in the hierarchical measures database would be applied in outpatient.

### Management practice of the respiratory infection control method in outpatient

The period of management practice in the outpatient department began on 1st September, 2021, and ended on 1st September, 2022. During this time, there was an average of 8000 outpatient visits per day. Under the strict supervision of the hospital infection management division and the outpatient management department, we performed weekly ILI and BI data prediction, evaluated the respiratory infection risk in outpatient setting by the risk assessment criteria and the predicted results, and implemented infection control measures from the hierarchical measures database according to the risk evaluation results. The number of HCWs who contracted COVID-19 from close contact with infected patients was regarded as a key indicator of the efficacy of infection control.

## Results

### Data collection and descriptive analysis

The Outpatient Management Department collected weekly data on all patients with ILI from doctors in the emergency department, fever clinic, and internal medicine clinic. The maximum number of patients with ILI was 331 during the second week of 2022, the minimum number was 16 during the 48th week of 2022, and the average number per week was 140.4.

A review of the literature identified 54 relevant articles, seven standards and two guidelines. Following data extraction, the research panel selected 24 infection control measures during high risk periods, including involving sterilization, 12 involving personal protection and five general suggestions.

### Model fitting and risk forecasting

Ergodic analysis showed that the ARIMA models 1,0,1; 1,1,1; 2,0,2, and 3,0,3 had the highest probability of achieving better model fit of the ILI and BI data.

**Fig. 1:**
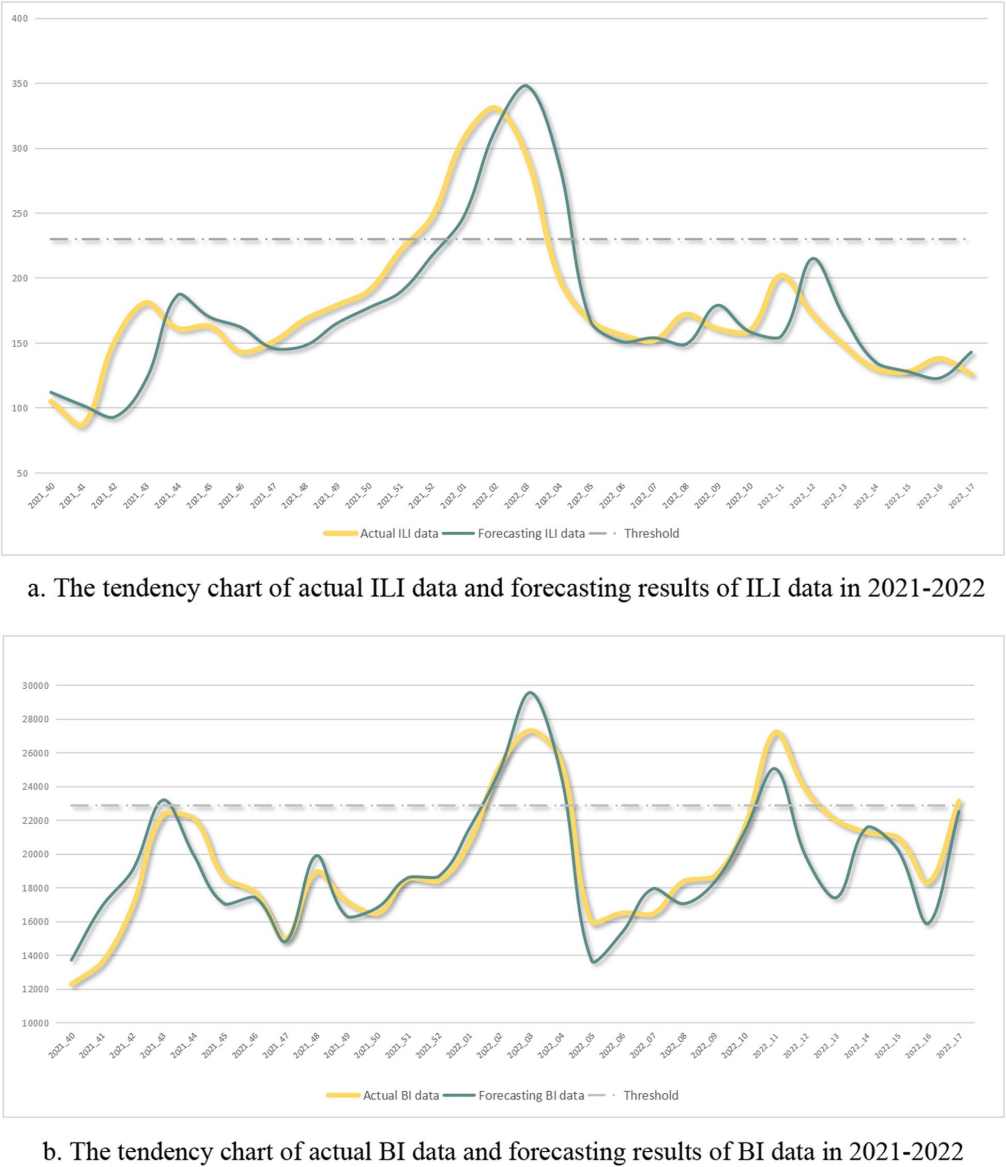
The tendency chart of ILI and BI data in 2021–2022

**Fig. 2:**
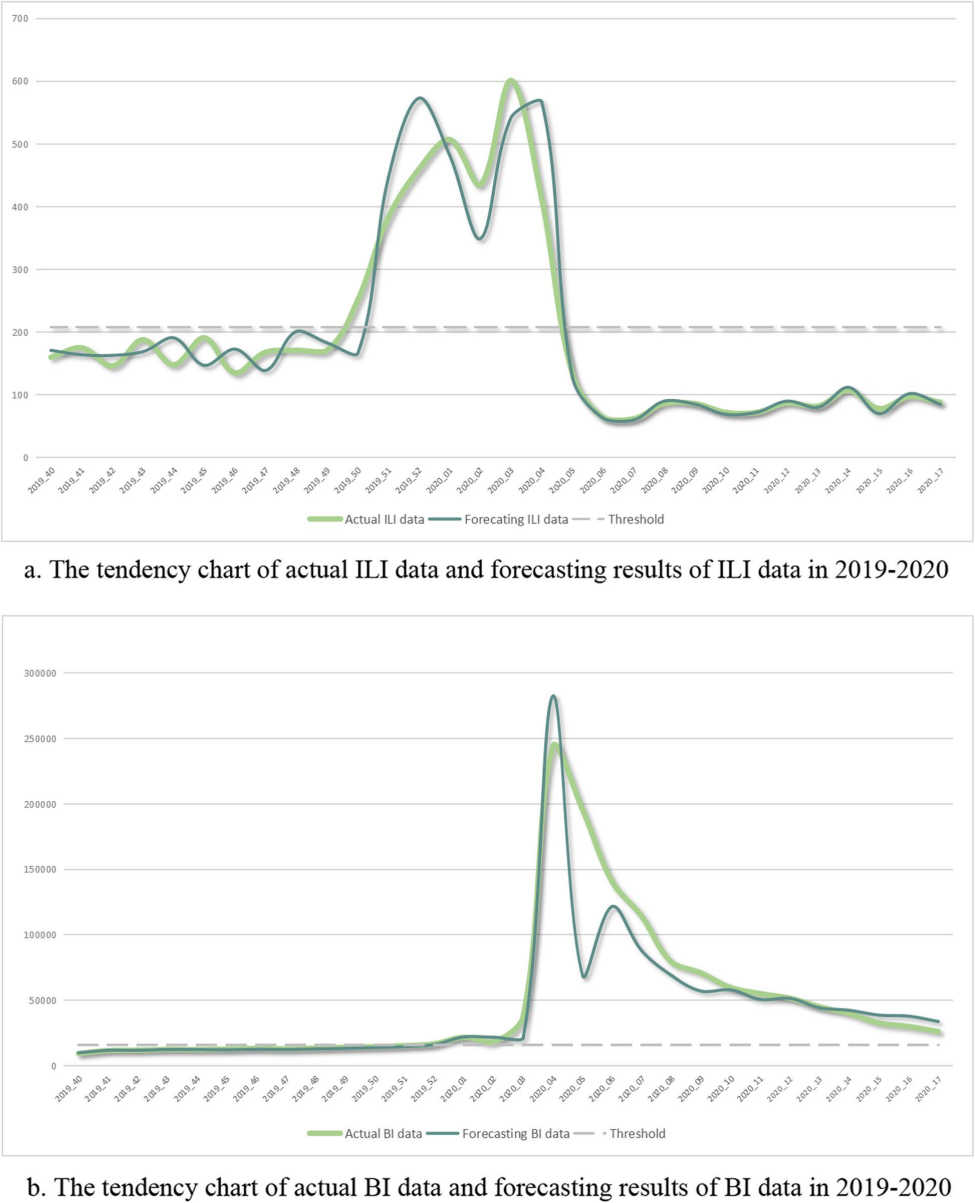
The tendency chart of ILI and BI data in 2019–2020

The rolling prediction results and the actual values of the two time period, 8th September 2019 to 1st May 2020, and 1st August 2021 to 31st December 2022, were showed in Figs. 1 and 2. The actual data was represented by the blue lines, the prediction results were represented by the green lines, and the thresholds dividing low and high risk levels were represented by gray lines. The MAPE results of the ILI and BI data during 1st August 2021 to 31st December 2022 were 13.65% and 8.04%, respectively.

### Hierarchical infection prevention measures and adjustment criteria

The hierarchical infection control measures are shown in Tables 1 (sterilization) and 2 (personal protection). In addition, the experts agreed on the following recommendations for infection control management during high-risk periods: 1) field or video inspections of outpatients and emergency room patients at least twice weekly; 2) making a list of recommend improvements for each hidden hazard or flaw found during inspection; 3) close monitoring and documentation of the health of patients who frequently visit the radiation oncology and hemodialysis departments; 4) provision of medical masks to outpatient visitors who exhibit symptoms of a respiratory tract infection; and 5) COVID-19 vaccination or booster injections of HCWs in the outpatient and emergency departments who do not have contraindications to the vaccine.

**Table 1:** The hierarchical infection prevention measures for sterilization

Locations		work cap		surgical mask	protective mask		medical protective clothes			isolation gown		face shield/ protective glasses		gloves		shoe cover
Low risk period																
Preview triage (excluded emergency and fever clinics)		-		+	±		-			-		-		-		-
Normal consulting room		+		+	-		-			-		-		-		-
Normal examination room		+		+	-		-			-		-		-		-
High risk consulting room		+		±	±		-			-		-		-		-
High risk examination room		+		±	±		-			±		-		+		-
Emergency outpatient (including preview triage)		+		+	±		±			±		-		±		±
Fever clinic (including preview triage)		+		±	±		±			-		±		±		±
Aerosol generating procedures		+		-	+		-			+		+		±		-
Medical waste collection		+		+	-		-			±		-		+		±
High risk period																
Preview triage (excluded emergency and fever clinics)	+		-			+		-	±		-		±		-	
Normal consulting room	±		±			±		-	-		-		-		-	
Normal examination room	±		±			±		-	** ± **		-		±		-	
High risk consulting room	+		-			+		-	-		-		-		-	
High risk examination room	+		-			+		-	±		-		+		-	
Emergency outpatient (including preview triage)	+		-			+		±	±		-		-		±	
Fever clinic (including preview triage)	+		-			+		±	±		±		-		±	
Aerosol generating procedures	+		-			+		±	±		+		+		-	
Medical waste collection	+		-			+		±	±		±		+		±	

1.High risk consulting room including: respiratory medicine, stomatology department and otolaryngology department

2.High risk examinatioin room including:high risk examinatioin room, Helicobacter pylori test and endoscopy

**Table 2:** The hierarchical infection prevention measures for personal protection

Locations	Low risk period				High risk period			
	Surrounding surface		Air		Surrounding surface		Air	
	Method	Frequency	Method	Frequency	Method	Frequency	Method	Frequency
Preview triage (excluded emergency and fever clinics)	☼	3	NV	3	☀	4	UV	1
Normal consulting room	☼	2	NV	2	☼	3	NV	3
Normal examination room	☼	2	NV	2	☼	3	NV	3
High risk consulting room	☼	3	NV	3	☀	3	UV	2
High risk examinatioin room	☼	3	NV	3	☀	3	UV	2
Emergency outpatient (including preview triage)	☀	3	NV	3	☀	4	UV	1
Fever clinic (including preview triage)	☀	3	UV	2	☀	4	UV	2
Medical waste collection	☀	3	NV	3	☀	3	NV	3

"☼" stands for 500mg/L Chlorine-containing disinfectant, "☀" stands for 1000mg/L Chlorine-containing disinfectant, “*UV*” stands for disinfection by ultraviolet light, “*NV*” stands for natural ventilation

The risk assessment criteria for starting to use high-risk prevention and control measures included: 1) an upward trend in actual ILI data during the previous two weeks (primary indicator); 2) the threshold being met or exceeded by two consecutive actual ILI data values (primary indicator); 3) the predicted BI data values meeting or surpassing the threshold (secondary indicator). The risk assessment criteria for starting to use low-risk prevention and control measures included: 1) a downward trend in actual ILI data during the previous two weeks (primary indicator); 2) one actual ILI value and the following predicted ILI value were below the threshold (primary indicator); or 3) both the actual and following predicted BI data values were below the threshold (primary indicator). In addition, due to the sharp increases in ILI data at the onset of COVID-19-related unexplained pneumonia, the panel agreed that infection control measures should be enhanced when actual ILI data increased more than 50% relative to the previous week.

### Management practice

The actual ILI data started climbing during week 46 of 2021 and reached the threshold during week 50. The forecasted result in the week 51 also reached the threshold. Similarly, the actual BI value reached the threshold during week 51 of 2021, strongly suggesting the need for high-level prevention measures. The COVID-19 infection control committee believed that infection risk would peak soon afterward and decided to upgrade infection prevention measures. When the ILI and BI data met the criteria during the week 6 of 2022, the committee decided to reduce infection prevention measures.

Of the 66 HCWs identified as having been in close contact with COVID-19 patients in outpatient clinics and emergency rooms during this period, none contracted COVID-19. In addition, nearly 41% of the cost of maintaining high-level infection prevention measures was saved by promptly adjusting personal protective equipment in outpatients and emergency room patients, in accordance with forecasted results.

## Discussion

In this study, we developed a novel risk management method for COVID-19 infection in outpatients. We found that with the estimation of ILI and BI data by ARIMA model and the hierarchical infection control measures. This management method can provide more efficient infection protection for healthcare workers and also improved the efficiency and accuracy of the infection control in outpatient settings.

### The fitting and application of ARIMA

The role of the prediction by ARIMA model in this study was a quantitative description for the respiratory infection risk in outpatient settings in the following week, and provide provides a basis for risk-based infection prevention and control in advance. Forecasting infection risks may improve the quality of infection control in outpatient settings. Several recent studies have focused on COVID-19 forecasting trends worldwide [39–41]. Using actual ILI and BI data, the rolling forecasting method utilized in this study performed satisfactorily, allowing infection prevention measures to be adjusted based on forecasting results. Because the time points at which ILI or BI data fell above or below the warning threshold played a crucial role in adjusting infection control measures, the ARIMA model can be better used to estimate the start rather than the peak or duration of an epidemic.

In addition, the rolling forecasting process did not maintain constant values for the model parameters p, d, and q. Multiple model fitting and testing showed that the optimum ranges for p and q were 1 to 4, whereas d was either 0 or 1. Therefore, use of the Ljung-Box test, AIC value, and acf and pacf graphs prior to each forecast may optimize high-quality forecasting.

The confidence of respiratory infection risk forecasting may be increased by the BI forecasting trend. Although these forecasts differed significantly from the actual data, the ARIMA model was able to identify early disease clusters or outbreaks [15] because their trends were in the same direction. Trends of ILI and BI data for COVID-19 were similar at the end of 2019 and the beginning of 2020, indicating the need for hospitals to adjust infection prevention measures. The actual ILI and BI data showed different trends after the sixth week, perhaps due to public responses and attention. These differences in trends also indicated the need for additional methods to better utilize and comprehend the forecasting results. However, pandemic results in 2020 and 2021 also showed that the forecast quality was high during these periods.

### Expert consensus on infection prevention in outpatients

The role of the expert consensus on infection prevention was like a “weapons depots”. It can provide the most appropriate respiratory infection control measures in outpatient settings to control different levels of COVID-19 infection risk in different time period, and save more cost. Expert consensus was based on a Delphi process that included two management sections, personal protection and disinfection. According to the key points for control of respiratory nosocomial infections, various infection prevention measures and personal protective equipment (PPE) were arranged in various areas or given to HCWs, such as aerosol generating procedures (AGPs) in the stomatology department [42]. To determine the linkage between the outcomes of risk forecasting with the consensus, low-risk and high-risk periods were considered independently.

Chlorinated disinfectants have been shown effective in killing viruses on various surfaces, leading to their widespread use [43]. Increases in the risk of infection have led to increases in the concentrations of chlorinated disinfectants and the frequency of wiping. Natural ventilation, the most efficient method of air sterilization, has been recommended in most low-risk outpatient areas [44]. In high-risk areas, such as dining and locker rooms, ultraviolet (UV) light should be used as the final disinfectant [45], with aerosol spray disinfection used during high-risk periods as a terminal disinfectant. Use of these disinfectants was not included in recommended disinfection measures, because these agents have a limited dispersion capacity and their application takes a long time. Although these disinfectants can thoroughly disinfect rooms with furniture or equipment, they are unable to keep pace with areas of outpatient care. Standard hand washing may be effective after contact with each infected person.

Standard precautions for nosocomial infection control and prevention should be applied to outpatients [46], with PPE selected based on experience with COVID-19 prevention measures. Close attention should be made to the selection of facial masks and to restricting the application range of medical protective clothing or isolation gowns, thereby preventing unexpected contamination from these overused PPE.

### The risk assessment criteria

ILI and BI data were found to have satisfied prediction performances in influenza epidemic trend [24, 33]. In this study, the risk assessment criteria was designed to act as a judgement method to analyze the level of respiratory infection risk that outpatient settings may face in the following week, according to the predicted value of ILI and BI data and the value range delimit.

Weekly ILI data was selected to be an indicator of actual infection risk in outpatients and emergency, while BI data was selected to be an indicator of the transmission intensity of respiratory infectious diseases in the community. ILI data can more accurately reflect the imminent risk of respiratory infections in outpatients, usually reaching thresholds and epidemic peaks earlier than BI data. So we selected the ILI data as primary indicator of the risk assessment criteria. The decrease of search engine index, like BI data, was associated with the reduction of community transmission pressure of respiratory infectious diseases [47]. It was suggested that BI data better reflected the transmission pressure on the community but not the pressure in outpatient, and was more suggestive of the downward trend. So we selected the BI data as primary indicator in the criteria of starting to use high-risk prevention and control measures, and as secondary indicator in the criteria of starting to use low-risk prevention and control measures.

### Study strengths and weaknesses

Use of the predicted results of an ARIMA model to guide infection control in outpatients was the most significant strength of this study. This model connected the requirements of infection control in outpatients with hierarchical infection control measures. In addition, this study used actual ILI surveillance data from an individual hospital, Xuanwu Hospital, better reflecting the infected patients in that hospital than data from the entire city of Beijing. Thus, the infection control measures suggested by this study may have greater applicability to patients at Xuanwu Hospital than to patients at other centers, including those in Beijing.

ILI and search engine data have been successfully utilized to predict the epidemic intensity of respiratory infectious diseases, suggesting the accuracy of these data. Although these data are easy to obtain, the effects of population movement, vaccination and other factors on disease transmission may be easily ignored, thus limiting data selection in the present study. Obtaining large sets of epidemiological data, such as those on population movement and vaccination, is difficult, indicating a need for multi-center studies or government-led research, as well as deep learning or artificial intelligence to provide more accurate predictions.

Because Xuanwu Hospital is a large general hospital, the infection prevention and control measures identified in the present study are likely applicable to large general hospitals. These measures are likely less applicable to patients in specialized hospitals, such as pediatrics, oncology, and stomatology hospitals, which may require more specialized prevention and control measures. Other limitations of this study include questions on practicality. For example, the model was written in R language, requiring the user to have some foundation in R language. There was no intelligent linkage between predicted results and infection control measures, indicating that these measures cannot be automatically implemented based on the predicted results. These drawbacks reduce the convenience of this novel infection control method. Additional studies are needed to design a novel comprehensive infection control system for various sized hospitals, as well as its incorporation into automated decision-making systems.

## Data Availability

All data generated or analyzed during this study are included in this published article.
